# Bad Hair Days: A Clinical and Trichoscopic Evaluation of Scalp Lesions

**DOI:** 10.7759/cureus.85265

**Published:** 2025-06-02

**Authors:** Ajeeth Rajkumar, Krishnakanth Muralidhar, Sai Preethi P., Adikrishnan Swaminathan, Murugan Sundaram, Sudha Rangarajan

**Affiliations:** 1 Dermatology, Sri Ramachandra Institute of Higher Education and Research, Chennai, IND

**Keywords:** alopecia, dermoscopy, scalp dermatoses, trichology, trichoscopy

## Abstract

Background

Scalp lesions encompass a diverse array of dermatological conditions, ranging from benign and transient issues to chronic, relapsing, or potentially scarring diseases. Accurate and timely diagnosis is crucial for appropriate management and prognostication. Traditional clinical examination, though essential, may not always suffice in differentiating between closely related scalp conditions. In this context, dermoscopy has emerged as a vital, non-invasive diagnostic adjunct. It provides enhanced visualization of subsurface skin and hair structures, aiding in pattern recognition and improving diagnostic accuracy for a variety of scalp disorders.

Aim

To evaluate and classify various scalp lesions based on clinical and trichoscopic features using a dermoscope.

Methods

A cross-sectional observational study was conducted over 20 months (August 2022 to March 2024) at a tertiary care centre. A total of 200 patients were enrolled. All patients underwent detailed clinical evaluation followed by trichoscopic examination using the DermLite DL4 dermoscope (10× magnification, polarized mode). Trichoscopic features were systematically recorded and correlated with clinical diagnoses and were grouped into major diagnostic categories and analyzed to identify characteristic patterns.

Results

A total of 200 were included in the study, with the highest prevalence observed in the 19- to 30-year age group. The most commonly reported symptoms were itching (64.5%) and hair loss (55%). The most frequent diagnostic categories were papulosquamous disorders (39%), alopecia (31.5%), infections and infestations (16%), autoimmune conditions (8.5%), and miscellaneous disorders (5%). Psoriasis (19%) and seborrheic dermatitis (17.5%) were the most prevalent individual diagnoses, while alopecia areata accounted for 12.5% of the cases. Trichoscopic examination in psoriasis patients revealed red dots as the most common feature (92.3%), followed by silvery-white scales (84.6%), nonspecific red areas (69.2%), and the “hidden hair” sign (61.5%). Abnormal vascular patterns (25.6%), yellow dots (20.5%), and perifollicular scaling (12.8%) were less frequent. In seborrheic dermatitis, the predominant trichoscopic feature was perifollicular scaling (65.7%), followed by atypical vascular patterns (42.8%, n=15), yellow dots and interfollicular scaling (40%) each, the “hidden hair” sign (37.1%), nonspecific red areas (34.2%), and red dots (8.5%). For alopecia areata, trichoscopic findings included black dots (84%), yellow dots (72%), vellus hairs (60%), broken hair shafts (44%), and the exclamation mark sign (28%).

Conclusion

Trichoscopy proved to be an invaluable, non-invasive diagnostic tool in the assessment of scalp lesions. It allowed rapid visualization of hallmark features, improving diagnostic accuracy and often reducing the need for invasive procedures such as scalp biopsies. The study demonstrates that distinct trichoscopic features serve as reliable markers for differentiating papulosquamous disorders and various forms of alopecia. Additionally, trichoscopy was effective in distinguishing between scarring and non-scarring alopecia, thereby enhancing diagnostic precision. Incorporating dermoscopy into routine dermatological evaluation improves diagnostic confidence and enables earlier, targeted interventions. Given its ease of use, reproducibility, and diagnostic value, trichoscopy should be considered an essential component in the evaluation of scalp dermatoses.

## Introduction

The scalp represents a distinct anatomical and physiological site, characterized by a high density of hair follicles and sebaceous glands compared to other regions of the body. Hair on the scalp serves multiple roles, including thermoregulation, protection against ultraviolet radiation, sensory perception, and contributing to social and aesthetic identity [1].

This unique microenvironment - dense, dark, and moist - is rich in sebum and cellular debris, creating a niche for diverse microbial communities, particularly around the follicular infundibulum. This region is actively monitored by antigen-presenting cells and various leukocyte populations within the epidermis. In contrast, the underlying dermis contains immune-privileged zones, specifically around the hair follicle bulge and bulb regions, which are critical for hair follicle cycling and regeneration. Disturbances in the local microbiota, destruction of immune-privileged sites, and pro-inflammatory responses precipitated by infections, stress, diet, local trauma, genetics, and epigenetic modifications can contribute to a spectrum of scalp dermatoses. These pathological changes often manifest as hair loss, thinning, scaling, pruritus, and scarring, frequently leading to significant psychological distress in affected individuals [2].

Scalp dermatoses may present with overlapping clinical features, complicating diagnosis. Scaling is a prominent feature in conditions such as psoriasis, seborrhoeic dermatitis, and tinea capitis. Patchy alopecia may be observed in trichotillomania and alopecia areata, while diffuse hair shedding is characteristic of telogen effluvium and androgenetic alopecia. Additionally, scarring and irreversible hair loss may occur in chronic inflammatory disorders such as lichen planopilaris, discoid lupus erythematosus, and morphoea.

Dermoscopy is a handheld, non-invasive diagnostic instrument that provides magnification ranging from 10× to 1000×. It is widely utilized in outpatient settings to facilitate rapid diagnosis, enable early therapeutic intervention, and potentially reduce the need for invasive procedures such as biopsies. Additional advantages include targeted examination of specific scalp regions, the use of polarized light to visualize deeper skin and follicular structures, and the capability to capture and store high-resolution images for longitudinal assessment and expert review [3].

The present study aims to diagnose, classify, and characterize trichoscopic patterns associated with various scalp disorders, while also evaluating the prevalence of these conditions among patients presenting to a tertiary care center.

## Materials and methods

This prospective cross-sectional study was conducted in the Department of Dermatology, Venereology, and Leprosy at a tertiary care hospital (Sri Ramachandra Medical College, Sri Ramachandra Institute of Higher Education and Research, Chennai) in South India over a two-year period (August 2022 to March 2024). The study was approved by the Institutional Ethics Committee (CSP-MED/22/AUG/78/81). A total of 200 individuals with previously undiagnosed and untreated scalp lesions were enrolled after obtaining written informed consent.

A detailed history was taken, followed by a clinical examination in a well-lit room. Dermoscopic examination was then performed on the entire scalp, using the DermLite DL4 dermoscope (3Gen, San Juan Capistrano, CA, USA) with a mobile phone attachment. Photographs were captured in both polarized and non-polarized modes. Contact mode was used for non-infectious cases, while non-contact mode was employed for infectious cases. The obtained photographs were reviewed and analyzed, and the findings were categorized.

Clinical diagnosis was primarily established based on patient history, physical examination, and dermoscopic assessment. When clinically indicated, supplementary diagnostic techniques such as trichogram analysis, Wood’s lamp examination, and potassium hydroxide (KOH) mount were also utilized. In cases suggestive of scarring alopecia, a scalp biopsy was performed for histopathological confirmation.

Data were entered into and analyzed using IBM SPSS Statistics for Windows, Version 26 (Released 2020; IBM Corp., Armonk, New York, United States).

## Results

A total of 200 patients were included in the study. The mean age at presentation was 27 ± 15.4 years, with the majority of patients falling within the 19- to 30-year age group. The study population comprised 107 males and 93 females, yielding a male-to-female ratio of 1.15:1. Symptomatically, the most common complaint was pruritus, reported by 64.5% of patients, followed by hair fall (55%) and scalp scaling (47%). Based on clinical and trichoscopic findings, the most prevalent category of scalp lesions was papulosquamous disorders, observed in 78 patients (39%). Alopecia was the second most common category, accounting for 63 cases (31.5%). Infectious and parasitic scalp conditions were identified in 32 patients (16%), while autoimmune disorders affecting the scalp were observed in 17 patients (8.5%). Additionally, 10 patients (5%) presented with miscellaneous scalp lesions and hair shaft abnormalities (Table 1).

**Table 1:** Distribution of scalp dermatoses among the study population (n=200)

Dermatoses	Number of Cases (n)	Percentage (%)
Psoriasis	39	19.50
Seborrheic dermatitis	35	17.50
Alopecia areata	25	12.50
Pediculosis	17	8.50
Androgenic alopecia	14	7
Folliculitis	8	4
Vitiligo vulgaris	7	3.50
Telogen effluvium	7	3.50
Discoid lupus erythematosus	6	3
Lichen planopilaris	5	2.50
Pemphigus vulgaris	4	2
Tinea capitis	4	2
Contact dermatitis	4	2
Acne keloidalis nuchae	3	1.50
Verrucae vulgaris	3	1.50
Trichotillomania	3	1.50
Pemphigus foliaceus	2	1
Bullous pemphigoid	2	1
Systemic lupus erythematosus	2	1
Miscellaneous	10	5
Total number of cases	200	100

Papulosquamous disorders

**Figure 1:**
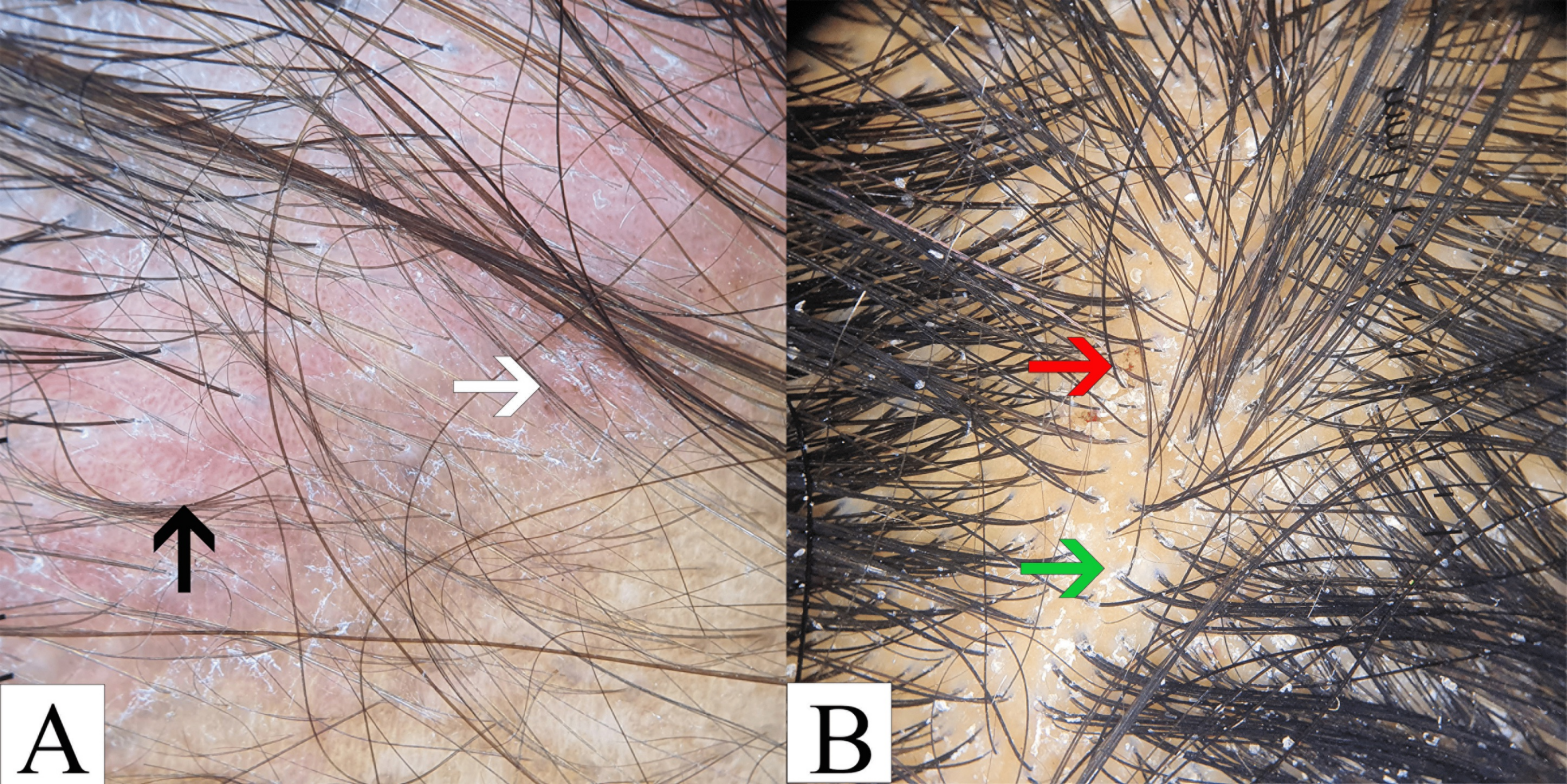
Dermoscopic (10x) image of (A) psoriatic plaque showing large white scales and abnormal vessels and (B) seborrhoeic dermatitis with perifollicular scaling and red dots Panel A Black arrow: abnormal blood vessels; white arrow: red dotted vessels Panel B Red arrow: red dots; green arrow: perifollicular scaling

In this study, psoriasis vulgaris emerged as the most frequently diagnosed scalp dermatosis. Affected individuals were predominantly within the 19- to 30-year age group, with a male-to-female ratio of 1.6:1. The majority of these patients presented with chronic plaque-type psoriasis with scalp lesions. Trichoscopic examination revealed red dots as the most prevalent dermoscopic feature, observed in 92.3% of patients (n=36). Silvery-white scales were present in 84.6% (n=33), followed by nonspecific red areas in 69.2% (n=27). The "hidden hair" sign was identified in 61.5% of cases (n=24). Abnormal vascular patterns were noted in 25.6% (n=10), yellow dots in 20.5% (n=8), and perifollicular scaling in 12.8% of patients (n=5) (Figure 1A).

Seborrhoeic dermatitis was the second most frequently diagnosed scalp condition, identified in 35 out of 200 patients. The majority of affected individuals were within the 19- to 30-year age group, with a male-to-female ratio of 1.18:1. Trichoscopic evaluation revealed perifollicular scaling as the most common finding, present in 65.7% of cases (n=23). Atypical vascular patterns were observed in 42.8% (n=15), followed by yellow dots and interfollicular scaling, each seen in 40% of patients (n=14). The "hidden hair" sign was identified in 37.1% of cases (n=13), and nonspecific red areas were noted in 34.2% (n=12). Red dots were the least commonly observed feature, present in only 8.5% of cases (n=3) (Figure 1B).

The dermoscopic features of psoriasis and seborrhoeic dermatitis are summarized in Table 2.

**Table 2:** Dermoscopic findings in papulosquamous disorders

Pattern	Number	Percentage (%)
Psoriasis (n=39)		
Red dots	36	92.30
Interfollicular scaling	33	84.60
Red areas	27	69.20
Hidden hair	24	62
Atypical vessels	10	26
Yellow dots	8	20.50
Perifollicular scaling	5	12.80
Seborrhoeic dermatitis (n=35)		
Perifollicular scaling	23	65.70
Atypical vessels	15	43
Yellow dots	14	40
Interfollicular scaling	14	40
Hidden hair	13	37.10
Red areas	12	34.20
Red dots	3	8.50

Non-scarring alopecia

**Figure 2:**
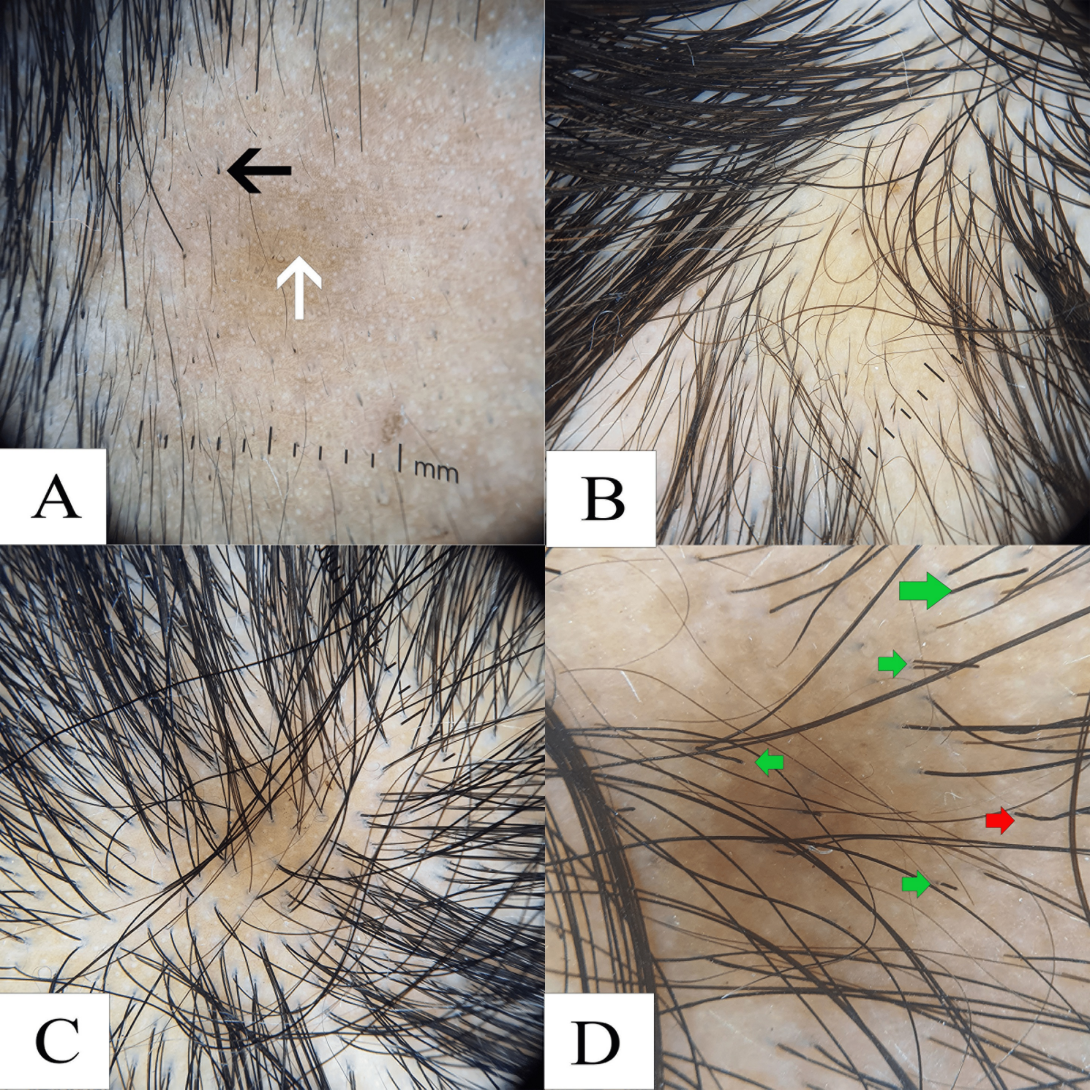
Dermoscopic (10x) image of (A) alopecia areata showing black dots, exclamation hair, and vellus hair; (B) Androgenic alopecia showing varied hair shaft diameter; (C) Telogen effluvium; (D) Trichotillomania showing hair shafts broken at different lengths and bent hair shafts Panel A Black arrow: exclamation hair; white arrow: black dots Panel D Green arrow: hair shafts of different lengths; red arrow: corkscrew hair

Alopecia areata was the most commonly diagnosed non-scarring alopecia in patients presenting with hair loss, affecting 25 individuals. The majority were aged between 19 and 30 years, with a male-to-female ratio of 1.08:1. Based on clinical presentation, single-patch alopecia areata was the most frequent subtype, followed by multiple-patch alopecia areata. Less common variants included ophiasis (n=4), alopecia totalis (n=2), and alopecia universalis (n=1). Trichoscopic findings revealed black dots in 84% of patients (n=21) and yellow dots in 72% (n=18). Vellus hairs were seen in 60% (n=15), broken hair shafts in 44% (n=11), and the exclamation mark sign in 28% (n=7) (Figure 2A).

Androgenetic alopecia was diagnosed in 14 patients, comprising 7% of the study population. Most were in the 19- to 30-year age group, with a male predominance (male-to-female ratio: 2.5:1). Trichoscopic examination showed hair shaft diameter diversity >20% in all patients (100%, n=14), and vellus hairs in 92.8% (n=13). Single-hair follicular units were noted in 57.1% (n=8), yellow dots in 42.8% (n=6), and the peri-pilar halo sign in 28.5% (n=4) (Figure 2B).

Telogen effluvium was observed in seven patients (3.5% of the total population), most of whom were females aged 19-30 years, with a male-to-female ratio of 0.4:1. Trichoscopy revealed single-hair follicular units in 57.1% (n=4), vellus hairs in 42.8% (n=3), peri-pilar halo in 28.5% (n=2), and yellow dots in 14.2% (n=1) (Figure 2C).

Trichotillomania was diagnosed in three patients. Dermoscopic features included broken hair shafts of varying lengths in all cases (100%), black dots in 100% (n=3), the V-sign in 66.6% (n=2), and perifollicular haemorrhage in one patient (33.3%) (Figure 2D).

The dermoscopic findings of non-scarring alopecia are tabulated in Table 3.

**Table 3:** Dermoscopic findings in non-scarring alopecia

Pattern	Number	Percentage (%)
Alopecia areata (n=25)		
Black dots	21	84.00
Broken hair	18	72.00
Vellus hair	15	60.00
Yellow dots	11	44
Exclamation hair	7	28
Androgenic alopecia (n=14)		
Hair diameter diversity >20%	14	100.00
Vellus hair	13	93
Single hair follicular unit	8	57.10
Yellow dots	6	43
Peripilar sign	4	29
Telogen effluvium (n=7)		
Single hair follicular unit	4	57.10
Vellus hair	3	42.80
Peri-pilar sign	2	28.50
Trichotillomania (n=3)		
Broken hair	3	100.00
Black dots	3	100.00
"V" sign	2	66.60
Perifollicular hemorrhage	1	33.30

Cicatricial alopecia

**Figure 3:**
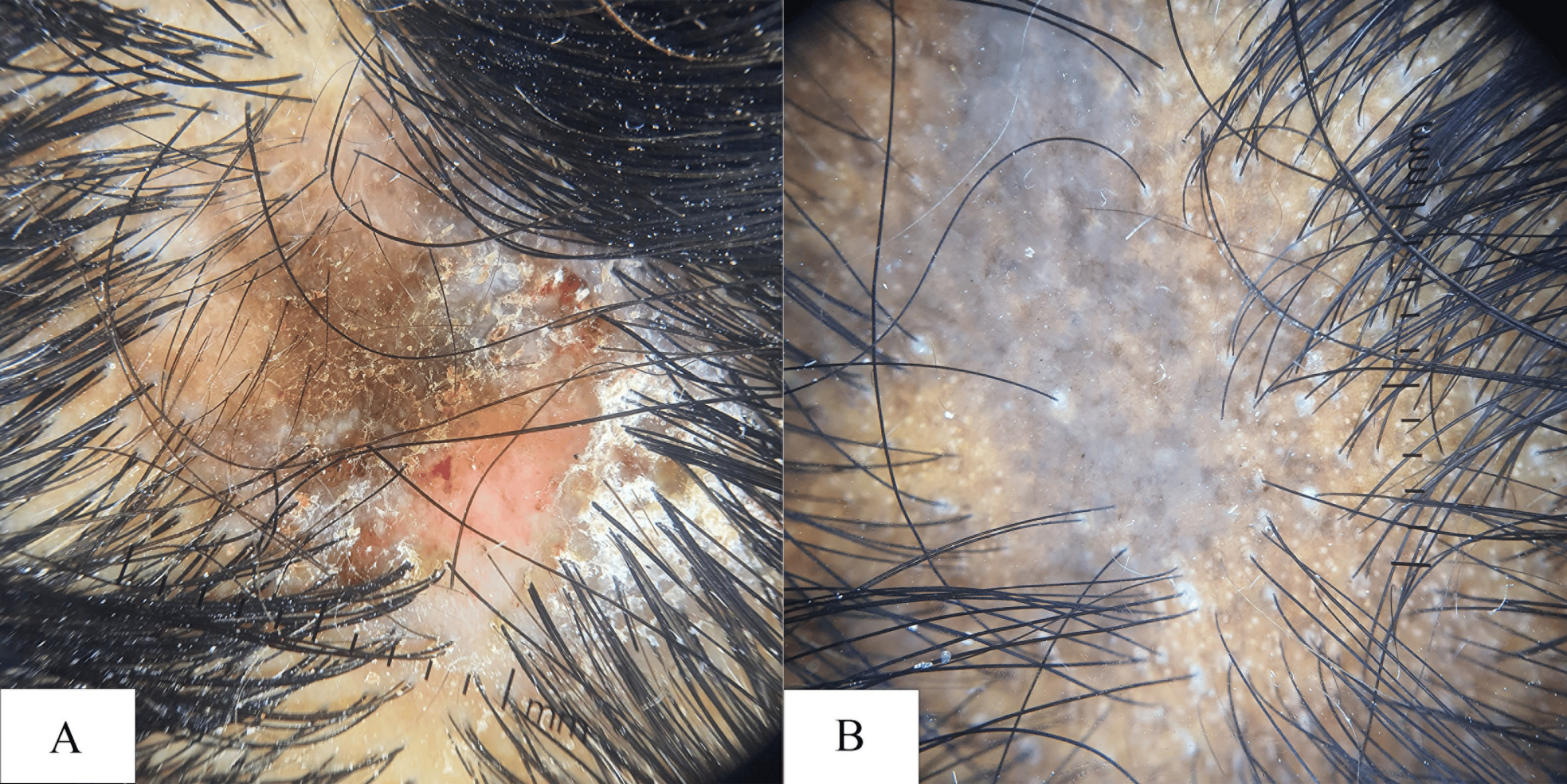
Dermoscopic (10x) image of (A) discoid lupus erythematosus and (B) lichen planopilaris

Discoid lupus erythematosus was the most frequently diagnosed cicatricial alopecia, seen in six female patients, primarily between the ages of 31 and 50 years. Trichoscopy revealed scarring of the follicular ostia in all patients (100%). Red dots were observed in 66.6% (n=4), white dots, aberrant vasculature, and follicular plugging were each seen in 50% (n=3). Erythema and perifollicular scaling were noted in one case each (16.6%) (Figure 3A).

Lichen planopilaris was diagnosed in five patients, with a male-to-female ratio of 0.66:1. Dermoscopy showed perifollicular scaling and follicular scarring were observed in all cases (100%, n=5). White dots were seen in 60% (n=3), erythema in 40% (n=2), and both red dots and aberrant vessels in 20% of cases (n=1 each) (Figure 3B).

The dermoscopic characteristics of cicatricial alopecia are summarized in Table 4. These findings aid in differentiating between various subtypes of scarring alopecia and provide essential clues for early diagnosis and management (Table 4).

**Table 4:** Dermoscopic findings in scarring alopecia

Pattern	Number	Percentage (%)
Discoid lupus erythematosus (n=6)		
Absent follicles	6	100
Red dots	4	66.60
Abnormal vessels	3	50
White dots	3	50
Follicular plugging	3	50
Erythema	1	16.60
Perifollicular scaling	1	16.60
Lichen planopilaris (n=5)		
Absent follicles	5	100
Perifollicular scaling	5	100
White dots	3	60
Erythema	2	40
Abnormal vessels	1	20
Red dots	1	20

Infections and infestations

**Figure 4:**
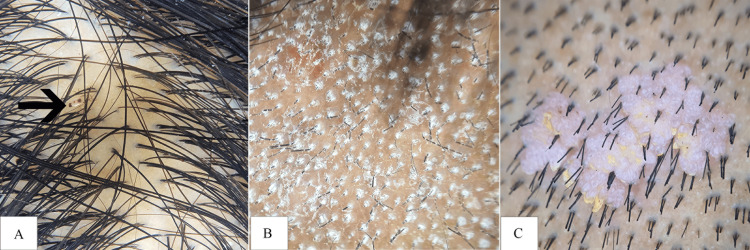
Dermoscopic (10x) images showing (A) pediculosis, (B) grey patch tinea, and (C) verrucae vulgaris Panel A Black arrow: *Pediculus humanus capitis*

Pediculosis capitis was the most frequently diagnosed scalp infestation, affecting 17 out of 200 patients, predominantly in individuals under 18 years of age. A marked female predominance was observed, with a male-to-female ratio of 0.13:1. Trichoscopic evaluation revealed the presence of nits in all affected individuals (100%), while live lice were visualized in 58.8% (n=10) (Figure 4A).

Tinea capitis was diagnosed in four patients. Of these, two presented with the grey patch variant (Figure 4B), and two with kerion. In the grey patch cases, dermoscopy demonstrated intense perifollicular scaling and broken hair shafts. In contrast, kerion lesions were characterized by marked erythema, follicular scarring, and the presence of pustules.

Three patients were diagnosed with verrucae vulgaris involving the scalp (Figure 4C). Dermoscopic examination revealed pinpoint haemorrhages in all cases (100%), and papillomatous surface changes in one patient (33.3%).

Autoimmune disorders

Among autoimmune disorders, vitiligo vulgaris was the most prevalent condition, affecting 3.5% (n=7) of the study population. The majority of individuals affected by vitiligo were in the 41 to 50 age range (42.8%, n=3), with a male-to-female ratio of 2.5:1. Dermoscopic findings in these cases revealed an altered pigmentary network in 71.4% (n=5) of patients, and leukotrichia in 42.8% (n=3).

Under immunobullous disorders with scalp involvement, pemphigus vulgaris was the most commonly observed condition, affecting 2% (n=4) of the study participants. The majority of these individuals were aged between 41 and 50 years. Dermoscopic features included extravasation (100%, n=4), crusting (75%, n=3), erythema (75%, n=3), and hair casts (50%, n=2). Pemphigus foliaceus, identified in 1% (n=2) of the cases, was exclusively observed in females within the 41 to 50 age group. Dermoscopic findings included extravasation (100%, n=2), crusting (100%, n=2), hair casts (100%, n=2), and erythema (50%, n=1). Bullous pemphigoid was also observed in 1% (n=2) of the population, affecting two females, with dermoscopic examination revealing crusting (100%, n=2), erythema (100%, n=2), and extravasation (100%, n=2).

**Figure 5:**
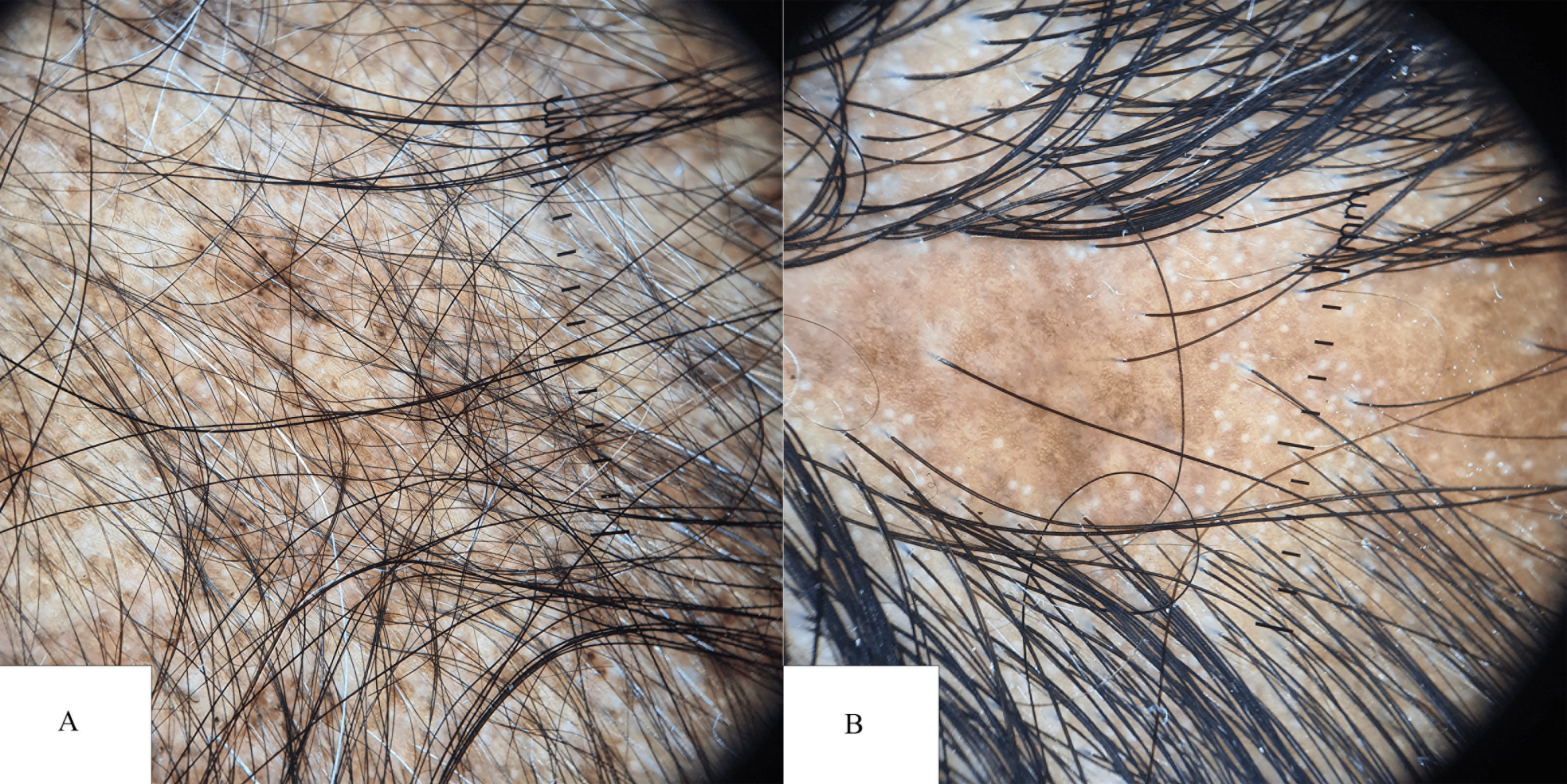
Dermoscopic (10x) images showing (A) systemic lupus erythematosus and (B) morphea

Systemic lupus erythematosus was diagnosed in two female patients. Dermoscopic findings for these cases included single hair follicular units (100%, n=2), abnormal vessels (100%, n=2), honeycomb brown pigmentation (100%, n=2), white dots (50%, n=1), and yellow dots (50%, n=1) (Figure 5A). A case of morphoea was diagnosed in a 24-year-old female, with dermoscopic features such as absent follicles, white dots, scaling, and the presence of a “hidden-hair” sign (Figure 5B).

Several other nonspecific scalp dermatoses were also documented in the study population. This included folliculitis (n=8), contact dermatitis (n=4), and acne keloidalis nuchae (n=3). Additional isolated conditions were recorded and are detailed in Table 5.

**Table 5:** Distribution of miscellaneous dermatoses among the study population (n=200)

Dermatoses	Frequency	Percentage (%)
Congenital triangular alopecia	1	0.50
Ichthyosiform erythroderma	1	0.50
Salmon patch	1	0.50
Griscelli syndrome	1	0.50
Naxos syndrome	1	0.50
Piebaldism	1	0.50
Nevus sebaceous	1	0.50
Oculocutaneous albinism	1	0.50

## Discussion

In the present study, the mean age at presentation was 27 years (SD ±15.4), with a wide age range spanning from six months to 75 years. The most frequently affected age group was 19 to 30 years, comprising 36.5% (n=73) of the total study population. In contrast, Aravamuthan et al. [4] reported a higher prevalence among individuals aged 0-18 years, while Bhat et al. [5] documented a slightly lower mean age of presentation at 24.87 ± 11.73 years.

A total of 200 patients were included in the study, with a male-to-female ratio of 1.15:1 (107 males (53%), 93 females (47%)), indicating a mild male predominance. This gender distribution is comparable to that reported by Aravamuthan et al. [4], who observed a male-to-female ratio of 1.8:1. In contrast, Bhat et al. [5] reported a higher proportion of female patients, with a ratio of 0.78:1.

Patients frequently presented with multiple overlapping symptoms. Pruritus was the most commonly reported symptom, affecting 64.5% of participants, followed by hair loss (55%), excessive scaling (47%), and pain (9%). These findings are consistent with those of Aravamuthan et al. [4], who identified itching (53.5%) as the predominant complaint, followed by hair fall (46.5%).

The clinical distribution of scalp dermatoses revealed that papulosquamous disorders were the most prevalent, accounting for 39% of cases. This was followed by alopecia-related disorders (31.5%), infections and infestations (16%), and autoimmune diseases (8.5%). A similar pattern was noted by Aravamuthan et al. [4], who reported papulosquamous conditions as the most frequent category (30.5%), followed by alopecia (27%).

Papulosquamous disorders

Psoriasis vulgaris emerged as the most prevalent papulosquamous disorder in this study, accounting for 19% (n=39) of the study population. The highest prevalence was observed in the 19-30 age group (30.7%, n=12), with a male-to-female ratio of 1.6:1. Chronic plaque psoriasis with scalp involvement was the predominant subtype (57%, n=22), followed by scalp psoriasis (28%, n=11). Dermoscopic evaluation revealed the presence of red dots in 92.3% (n=36) of cases, interfollicular scales in 84.6% (n=33), red areas in 69.2% (n=27), the hidden hair sign in 61.5% (n=24), and atypical vessels in 25.6% (n=10). These findings align with the study by Kibar et al. [6], although their cohort demonstrated a higher prevalence of atypical red vessels (67%).

Seborrheic dermatitis was the second most common diagnosis, affecting 17.5% (n=35) of participants, with the highest incidence also in the 19-30 age group. The male-to-female ratio was 1.18:1. Dermoscopic features of seborrheic dermatitis included perifollicular scaling in 65.7% (n=23) of cases, atypical vessels in 42.8% (n=15), yellow dots in 40% (n=14), the hidden hair sign in 37.1% (n=13), red areas in 34.2% (n=12), and red dots in 8.5% (n=3). These findings were comparable to those reported by Kibar et al. [6].

Contact dermatitis was diagnosed in 2% (n=4) of the patients, with the highest prevalence among individuals aged 41-50 years. Dermoscopic findings included scaling in all cases (n=4), erythema in 75% (n=3), abnormal vessels in 50% (n=2), and yellow dots in 50% (n=2). Starace et al. [7] similarly reported findings of 100% erythema and scaling, along with arborizing vessels in 91.2% of cases.

Additionally, a single case of pityriasis rubra pilaris was identified, showing broken hair shafts and perifollicular scaling on dermoscopy. These findings were consistent with those described by Zhou et al. [8].

Alopecia

Non-scarring alopecia was observed to be more prevalent than scarring alopecia in this study, with 77.8% of patients (n=49) presenting with non-scarring types, compared to 22.2% (n=14) with scarring forms. Among non-scarring alopecias, alopecia areata emerged as the most common subtype (n=25), with the highest incidence seen in the 19-30 age group (32%). The male-to-female ratio was 1.08:1. Dermoscopic examination commonly revealed black dots in 84% (n=21), yellow dots in 72% (n=18), vellus hairs in 60% (n=15), broken hair shafts in 44% (n=11), and exclamation mark hairs in 28% (n=7). Notably, these findings differ from those reported by Vijay et al. [9], where black dots were less commonly reported (36.5%).

Androgenetic alopecia accounted for 7% of cases (n=14), predominantly affecting individuals aged 19-30 years (78.5%, n=11), with a marked male preponderance (male-to-female ratio: 2.5:1). Dermoscopic features consistently included hair diameter variability greater than 20% (100%, n=14), vellus hairs (92.8%, n=13), single-hair follicular units (57.1%, n=8), yellow dots (42.8%, n=6), and peri-pilar halo (28.5%, n=4). These findings align with those reported by Bains et al. [10], who also observed a high prevalence of vellus hairs (100%) and prominent hair diameter variability >20% (51.6%) in androgenetic alopecia.

Telogen effluvium was diagnosed in seven patients, with a majority in the 19-30 age group (n=4). This condition was more common among females, with a male-to-female ratio of 0.4:1. Trichoscopic evaluation in these patients revealed single-hair follicular units in 57.1% (n=4) and vellus hairs in 42.8% (n=3). When compared to androgenetic alopecia, vellus hair was significantly less common in telogen effluvium, consistent with the observations of Bains et al. [10], who reported vellus hairs in only 12.1% of telogen effluvium cases.

Trichotillomania was identified in 1.5% of patients (n=3), with a majority belonging to the pediatric age group (n=2). Dermoscopic examination consistently revealed broken hairs and black dots in all cases (100%, n=3). Additional findings included the "V" sign in 66.6% (n=2) and perifollicular hemorrhage in one case (33.3%, n=1). These trichoscopic features are consistent with those described by Elmas and Metin [11], who similarly highlighted the diagnostic relevance of broken hairs, black dots, and perifollicular changes in trichotillomania.

Cicatricial alopecia

Among scarring alopecias, discoid lupus erythematosus (DLE) was the most frequently encountered condition, accounting for 1.5% of cases (n=6). All affected individuals were female and fell within the 31-50 age group. Dermoscopic findings in DLE included absent follicular openings (100%, n=6) and red dots (66.6%, n=4), with additional features such as white dots, aberrant vasculature, and follicular plugging were each seen in 50% (n=3), erythema (16.6%, n=1), and perifollicular scaling (16.6%, n=1). These observations are in concordance with the findings reported by Köse and Güleç [12], who emphasized the utility of dermoscopy in identifying hallmark features of DLE.

Lichen planopilaris (LPP) was diagnosed in five patients, predominantly within the 19-30 age group, with a slight female predominance (male-to-female ratio: 0.66:1). Dermoscopic examination demonstrated absence of follicular openings and peri follicular scaling in all cases (100%, n=5), along with white dots in 60% (n=3), erythema in 40% (n=2), both red dots and abnormal vascular patterns in 20% (n=1). These findings closely mirror those documented by Köse and Güleç [12], supporting the diagnostic role of dermoscopy in differentiating scarring alopecias.

Acne keloidalis nuchae was observed in 1.5% of the study population (n=3), primarily affecting males aged 31-40 years. Dermoscopic evaluation revealed erythema in all cases (100%, n=3), with pustules present in one case (33.3%). In comparison, a larger multicenter study conducted by Sánchez-Dueñas et al. [13] reported pustules in 48% of patients and erythema in 56.3%, indicating variability in clinical and dermoscopic presentation depending on disease stage and severity.

Infections and infestations

Among the infections and infestations, pediculosis was the most prevalent, affecting 8.5% (n=17) of the study population. The condition was predominantly observed in individuals under 18 years of age, who accounted for 82.3% (n=14) of the cases. The male-to-female ratio was 0.13:1. Dermoscopic examination revealed the presence of nits in all cases (n=17), while lice were identified in 58.8% (n=10) of cases. These findings were consistent with those reported by Nikam and Mehta [14].

Folliculitis was the second most common infestation, affecting 4% (n=8) of the study population, with individuals in the 19-30 and 31-40 age groups being the most affected. Dermoscopic features included erythema in all cases (n=8) and pustules in 75% (n=6) of the cases.

Tinea capitis was diagnosed in 2% (n=4) of the cases, primarily affecting children under 18 years of age. Dermoscopic findings included scaling in 100% (n=2) of cases, black dots in 50% (n=1), and corkscrew hair in 100% (n=2) of cases associated with grey patch tinea. These findings were in accordance with the study by Kumar et al. [15], who reported black dots as the most common dermoscopic finding, followed by scaling. In cases of scarring tinea capitis, absence of follicles (100%, n=2) and pustules (50%, n=1) were observed. Basurto et al. [16] reported similar dermoscopic features for kerion.

Verrucae vulgaris was observed in 1.5% (n=3) of patients, primarily affecting children under 18 years of age. Dermoscopic examination revealed pinpoint hemorrhages in all cases (100%), with one case showing papillomatosis growth. These findings were consistent with those reported by Sukanya et al. [17].

Autoimmune diseases

Vitiligo vulgaris emerged as the most common autoimmune disorder in this study, accounting for 3.5% (n=7) of the cases. The majority of affected individuals were in the 41-50 age group (42.8%, n=3), with a male-to-female ratio of 2.5:1. Dermoscopic features included an altered pigmentary network in 71.4% (n=5) and leukotrichia in 42.8% (n=3). These findings were consistent with those reported by Awal et al. [18].

Among immunobullous disorders, pemphigus vulgaris was the most frequently observed condition, affecting 2% (n=4) of the study population, predominantly in the 41-50 age group. Dermoscopic findings included extravasations (100%, n=4), erythema (75%, n=3), crusting (75%, n=3), and hair casts (50%, n=2). Pemphigus foliaceus was diagnosed in 1% (n=2) of cases, all of whom were females aged 41-50, with dermoscopic findings of hair casts (100%, n=2), crusting (100%, n=2) extravasations (100%, n=2), and erythema (50%, n=1). The study by Sar-Pomain et al. [19] similarly noted that extravasations were more common in pemphigus vulgaris, while hair casts were more frequently observed in pemphigus foliaceus. Bullous pemphigoid, observed in 1% (n=2) of the study population, affected two females and showed dermoscopic features such as crusting (100%, n=2), erythema (100%, n=2), and extravasations (100%, n=2).

Systemic lupus erythematosus (SLE) was noted in two females, with dermoscopic findings including single hair follicular units (100%, n=2), abnormal vessels (100%, n=2), honeycomb brown pigmentation (100%, n=2), as well as white (50%, n=1) and yellow (50%, n=1) dots. These findings are in line with those reported by Chanprapaph et al. [20]. Morphoea was diagnosed in a 24-year-old female, exhibiting absent follicles, white dots, scaling, and the hidden hair sign. These dermoscopic features were similarly observed in the study by Saceda-Corralo et al. [21].

Limitations

The size of the study population and the fact that it was obtained predominantly from a single tertiary care center may limit the ability to generalize the findings to a broader population. A multicentric study with a larger sample size would have enhanced the validity of the study for a larger population. Additionally, clinical findings derived from trichoscopy are subjective, and variations in the interpretation of dermoscopic images could influence the consistency and reliability of the results.

## Conclusions

The scalp and its appendages create a dark region where lesions may not be immediately visible to the naked eye, thus necessitating the need for specialized tools like dermoscopy. This study provides the prevalence, clinical presentation, and trichoscopic features of various scalp dermatoses observed at a tertiary care center. The findings emphasize the utility of dermoscopy as an effective non-invasive diagnostic tool in the evaluation of scalp conditions, enabling early diagnosis and management. Additionally, the study highlighted the importance of dermoscopy in differentiating between scarring and non-scarring alopecias, providing clarity in conditions like discoid lupus erythematosus and lichen planopilaris.

In conclusion, dermoscopy is a crucial diagnostic tool in scalp dermatology, offering significant advantages in terms of non-invasive evaluation, early detection, and minimizing the need for biopsies. The study emphasizes the need for continued exploration of the clinical and dermoscopic patterns associated with various scalp disorders to refine diagnostic strategies and enhance therapeutic outcomes.
